# Rubber oxygenases

**DOI:** 10.1007/s00253-018-9453-z

**Published:** 2018-10-30

**Authors:** Dieter Jendrossek, Jakob Birke

**Affiliations:** Institute of Microbiology, University of Stuttgart, Allmandring 31, 70550 Stuttgart, Germany

**Keywords:** Natural rubber, Poly(*cis*-1,4-isoporene), Rubber oxygenase, Latex clearing protein, RoxA, RoxB, Lcp

## Abstract

Natural rubber (NR), poly(*cis*-1,4-isoprene), is used in an industrial scale for more than 100 years. Most of the NR-derived materials are released to the environment as waste or by abrasion of small particles from our tires. Furthermore, compounds with isoprene units in their molecular structures are part of many biomolecules such as terpenoids and carotenoids. Therefore, it is not surprising that NR-degrading bacteria are widespread in nature. NR has one carbon-carbon double bond per isoprene unit and this functional group is the primary target of NR-cleaving enzymes, so-called rubber oxygenases. Rubber oxygenases are secreted by rubber-degrading bacteria to initiate the break-down of the polymer and to use the generated cleavage products as a carbon source. Three main types of rubber oxygenases have been described so far. One is rubber oxygenase RoxA that was first isolated from *Xanthomonas* sp. 35Y but was later also identified in other Gram-negative rubber-degrading species. The second type of rubber oxygenase is the latex clearing protein (Lcp) that has been regularly found in Gram-positive rubber degraders. Recently, a third type of rubber oxygenase (RoxB) with distant relationship to RoxAs was identified in Gram-negative bacteria. All rubber oxygenases described so far are haem-containing enzymes and oxidatively cleave polyisoprene to low molecular weight oligoisoprenoids with terminal CHO and CO–CH_3_ functions between a variable number of intact isoprene units, depending on the type of rubber oxygenase. This contribution summarises the properties of RoxAs, RoxBs and Lcps.

## Introduction

Natural rubber (NR) is produced mainly by plants and is the characteristic and main component of rubber latex particles. NR-producing species are frequently found in species of the *Euphorbiaceae*, e.g. in the rubber tree (*Hevea brasiliensis*) or in the members of the *Compositae* such as in *Taraxacum koksaghyz* (Russian dandelion) (van Beilen and Poirier 2007a, b). The ability to synthesise rubber can also be found occasionally in species of other plant families and even in some fungi. Most rubber-accumulating plants synthesise the polymer with the isoprene units in the *cis*-configuration, whereas some species such as *Manilkara chicle* or *Palaquium gutta* synthesise the *trans*-polymer, leading to rubbers known as chicle or gutta-percha. Poly(*cis*-1,4-isoprene) latex in *Hevea brasiliensis* and many other species is synthesised and accumulated in form of globules that can have a diameter of several hundreds of nanometres up to a few micrometres. The rubber particles are stored under pressure in special tissues (laticifers) and are released as latex after injury of the tissue, e.g. by the tapping process or during invading of insect larvae. Latex globules consist of a polyisoprene core that is covered by a phospholipid monolayer (Wadeesirisak et al. 2017; Cornish et al. 1999) into which several proteins such as hevein (Berthelot et al. 2016) and the polymer-synthesising enzyme (*cis*-polyprenyl-synthetase) are attached (Schmidt et al. 2010; Berthelot et al. 2014). For overviews on rubber biosynthesis and latex globule structure, see Cornish et al. (1999), Cornish (2001), Berthelot et al. (2014) and Epping et al. (2015). Defence against parasites could be one of the main functions for the production of polyisoprene latex given that latex released after damaging the laticifers rapidly coagulates and encapsulates the invading pest.

## Rubber-degrading bacteria

Since the invention of crosslinking the polyisoprene chains with sulphur (vulcanisation) by Goodyear in 1844, rubber has been permanently in use in a large industrial scale (currently ≈ 10^7^ tons NR per year). Despite the enormous amounts of rubber particles that are constantly released to the environment by abrasion from tires for a period of meanwhile more than a century, there is no evidence for a long-term accumulation of such rubber particles pointing to the ubiquitous presence and efficiency of rubber-degrading microorganisms. Indeed, rubber-degrading microorganisms have been identified in and isolated from most ecosystems with moderate physical parameters (temperature, pH, salinity). For lists and overviews on rubber-degrading bacteria, see Jendrossek et al. (1997), Rose and Steinbüchel (2005), Warneke et al. (2007), Yikmis and Steinbüchel (2012a), Chengalroyen and Dabbs (2013) and Shah et al. (2013). The majority of the currently known and well-characterised rubber-degrading bacteria are Gram-positives and belong to the actinobacteria such as species of the genera *Nocardia* (Tsuchii et al. 1985; Ibrahim et al. 2006; Linh et al. 2017), *Streptomyce*s (Heisey and Papadatos 1995; Jendrossek et al. 1997; Rose et al. 2005; Imai et al. 2011; Chia et al. 2014; Nanthini et al. 2017), *Gordonia* (Linos et al. 1999; Linos et al. 2002) and *Rhodococcus* (Watcharakul et al. 2016). Gram-negative rubber-degrading bacteria seem to be less abundant and only one Gram-negative strain with clearly demonstrated rubber-degrading ability was known until recently. This strain is *Steroidobacter cummioxidans* 35Y (Sharma et al. 2018) (previously *Xanthomonas* sp. 35Y (Tsuchii and Takeda 1990)) and meanwhile has become one of the best-studied rubber degraders (see below). In the present decade, other Gram-negative rubber-degrading species have been isolated and described such as *Rhizobacter gummiphilus* (Imai et al. 2010; Imai et al. 2013) or several species of the myxobacteria (Birke et al. 2013). Other Gram-negative rubber-degrading bacteria (*Pseudomonas aeruginosa*, *Pseudomonas citronellolis* and *Acinetobacter calcoaceticus*) as well as two rubber-degrading *Bacillus* strains (AF-666 and S10) have been also reported but the evidence for the ability to utilise polyisoprene as the sole source of carbon and energy is yet not fully convincing (Linos et al. 2000; Bode et al. 2000; Bode et al. 2001; Shah et al. 2012; Kanwal et al. 2015) or has been shown to be caused by a mixed culture of a true rubber-degrading *Gordonia* species and *P. aeruginosa* AL98 (Arenskötter et al. 2001).

### Identification of rubber-degrading bacteria

The majority of the currently described rubber-degrading bacteria produces translucent halos when cultivated on opaque latex-containing solid agar media. The formation of clear zones around the developing colonies of rubber degraders is a consequence of the secretion of rubber-degrading enzymes. These enzymes diffuse into the agar and extracellularly cleave the polymer to low molecular products that can be taken up by the bacteria. The formation of clearing zones indicates that the insoluble polyisoprene latex is converted to smaller products and that the cleavage products, which are also almost insoluble in water, are taken up by the cells and used as a carbon and energy source. Many rubber-degrading *Streptomyces* strains such as *S. coelicolor* 1A, *S. griseus* 1D and *Streptomyces* sp. K30 but also Gram-negative species such as *S. cummioxidans* 35Y (*Xanthomonas* sp. 35Y) and *R. gummiphilus* NS21 have the ability to form clearing zones on polyisoprene latex agar (Jendrossek et al. 1997; Braaz et al. 2004; Rose et al. 2005; Imai et al. 2011). Interestingly, other potent rubber degraders such as *Gordonia polyisoprenivorans* VH2, *Gordonia westfalica* Kb2, *Nocardia farcinica*, *Nocardia nova* SH22a and *Rhodococcus rhodochrous* RPK1 or *Rhodococcus pyridinivorans* F5 do not form clearing zones on latex agar (Ibrahim et al. 2006; Warneke et al. 2007; Bröker et al. 2008; Luo et al. 2014; Watcharakul et al. 2016; Nawong et al. 2018). Non-clearing zone-forming rubber degraders grow adhesively on the rubber surface and produce no visible clearing zone as shown by electron microscopy (Linos et al. 2000). Adhesively growing strains have specific Mce (mammalian cell entry) transmembrane substrate uptake systems for the incorporation of long-chain polyisoprene cleavage products (for details, see Luo et al. 2014).

### Rubber oxygenase RoxA of *Steroidobacter cummioxidans* 35Y

*S. cummioxidans* 35Y is the best-studied Gram-negative rubber degrader. Strain 35Y was first described by Tsuchii and Takeda in 1990 (Tsuchii and Takeda 1990) and the authors of this early study already described a “rubber-cleaving activity” in the supernatant of latex-grown 35Y cells and identified aldehyde and keto groups containing cleavage products. Unfortunately, the ecosystem from which strain 35Y was isolated is not known anymore. The only information on the source of the strain found in the publication is “Bacterial strain 35Y, a potent producer of rubber-degrading enzyme, was selected from our culture collection” (Tsuchii and Takeda 1990).

The enzyme responsible for the initial cleavage of rubber by strain 35Y was isolated and biochemically characterised and the corresponding structural gene was cloned and sequenced (Jendrossek and Reinhardt 2003). The rubber-cleaving enzyme of strain 35Y is a 70-kDa dihaem protein that oxidatively cleaves poly(*cis*-1,4-isoprene) to the C_15_ compound, 12-oxo-4,8-dimethyltrideca-4,8-diene-1-al (ODTD), as the only major end product (Braaz et al. 2004). The protein was denominated as rubber oxygenase A (RoxA). The two haem groups in RoxA are covalently attached to the apoprotein (*c*-type haem) via thioether bridges of cysteine sulphur atoms of two haem binding motifs (CxxCH) to the propionate side chains of the haem-porphyrin. The cleavage of polyisoprene to only one major product (ODTD) indicates that RoxA acts processively and cleaves rubber from one end of the polyisoprene chain in an *exo*-type fashion. ^18^O_2_-labeling experiments revealed that RoxA is a dioxygenase (Braaz et al. 2005). The RoxA protein is stable (even at room temperature) and does not need any additional cofactors. Water, adjusted to a neutral pH value, polyisoprene (purified natural rubber latex or synthetic polyisoprene) and the co-substrate dioxygen are the only compounds necessary for efficient cleavage of polyisoprene to ODTD (2.6 U oxygen consumption/mg protein at 37 °C, see Table 1). The two haem groups of RoxA have slightly different midpoint potentials (E_o_´) of − 65 mV and of − 130 to − 160 mV (not well resolvable) and in vitro react differently upon the addition of reductants (NADH) (Schmitt et al. 2010). The N-terminal haem group represents the active site and has a dioxygen molecule stably bound in the *as isolated* state (Seidel et al. 2013). Additional properties of the RoxA protein are discussed in the chapter of the structures of rubber oxygenases below.

**Table 1:** Properties of biochemically characterised rubber oxygenases

Protein/attribute	Rox_A35Y_	RoxA_NS21_	RoxA_Cco35_	RoxA_Mfu_	RoxA_Hoc_	RoxB_NS21_	RoxB_35Y_	Lcp_K30_	Lcp_RPK1_	Lcp_VH2_
Gene length [bp]	2037	2022	2022	2025	2031	2040	2046	1224	1227	1224
Secretion system*	Sec	Sec	Sec	Sec	Sec	Sec	Sec	Tat	Tat	Tat
Mw pre-protein [kDa]	74.7	74.4	73.9	74.6	75.1	74.0	73.8	44	45.2	45.5
Mw mature [kDa]	71.5	71.5	71.1	71.9	72.1	70.8	70.3	41	42.2	41.7
P_i_ mature (theor.)**	7.3	8.0	6.7	7.0	4.9	6.7	6.3	6.1	5.2	6.0
% of arom. AA [in mature protein]	11.4	11.4	11.3	11.6	12.7	10.5	9.9	7.7	9.0	9.7
(Total no.: F, Y, W)	(24, 30, 20)	(27, 25, 22)	(34, 23, 16)	(32, 27, 16)	(35, 30, 17)	(25, 25, 18)	(23, 24, 17)	(7, 10, 12)	(9, 11, 14)	(11, 12, 13)
Haem attachment										
N-terminal	CSACH_195_	CSACH_181_	CSGCH_182_	CSGCH_182_	CSGCH_181_	CHACH_196_	CHACH_196_	–	–	–
C-terminal	CASCH_394_	CASCH_379_	CASCH_380_	CASCH_380_	CASCH_379_	CASCH_395_	CASCH_395_	–	–	–
Axial haem ligands										
N-terminal	H_195_	H_181_	H_182_	H_182_	H_181_	H_196_	H_196_	H_198_ (K_167_)	H_197_ (K_166_)	H_195_ (K_164_)
C-terminal	H_394_ H_641_	H_379_ H_626_	H_380_ H_627_	H_380_ H_627_	H_379_ H_626_	H_395_ H_627_	H_395_ H_627_	–		
Fe state “as isolated”	Fe^2+^--O_2_/Fe^3+^	Fe^2+^--O_2_/Fe^3^+	Fe^2+^--O_2_/Fe^3+^	Fe^2+^--O_2_/Fe^3+^	Fe^2+^--O_2_/Fe^3+^	Fe^3+^/Fe^3+^	Fe^3+^/Fe^3+^	Fe^3+^	Fe^3+^	Fe^3+^
N-/C-terminal										
MauG motif	PYFH_517_ NGSVP	PFFH_502_ NGSVP	PFFH_503_ NGSVP	PYFH_503_ NSSVP	PYFH_502_ NGSVP	PYLH_494_ NGSVP	PYMH_494_ NGSVP	–	–	–
F_317_ equivalent	F_317_	F_302_	F_303_	F_303_	F_302_	F_309_	F_309_	–	–	–
W_302_ equivalent	W_302_	W_287_	W_288_	W_288_	W_287_	V_294_	V_294_	–	–	–
conserved DUF2236 residues (R/T/H)	–	–	–	–	–	–	–	164/168/198	163/167/197	161/165/195
Soret max (ox) [nm]	407	407	407	408	409	404	404	412	407	412
(Reduced) [nm]	418	417	418	418	418	418	419	430	428	430
Beta (reduced) [nm]	549, 553	549, ~ 551	549, 553	551–552	550, 553	548, 556	548, 556	562	562	564
Alpha (reduced) [nm]	521	522	522	522	523	522, 529	522, 529	532	532	533
Bands above 600 nm	No	No	No	No	No	~ 618	~ 618	No	~ 645	No
UVvis effect upon addition of CO	Yes	Yes	n.d.	n.d.	n.d.	No	No	No	No	No
Cleavage product(s)	ODTD	ODTD	ODTD	ODTD	ODTD	Pattern	Pattern	Pattern	Pattern	Pattern
Specific activity [U/mg] at 23, 30, 37 °C	0.9/1.9/2.6	0.1/n.d./n.d.	~ 10% of RoxA_35Y_	~ 10% of RoxA_35Y_	n.d.	4.8/n.d./n.d.	4.5/5.7/6.4	1.5/n.d./4.7	0.9/3.1/n.d.	1.3/n.d./n.d.

Lcps, positions in premature-native sequence

RoxAs, positions in mature sequence

*n.d.*, not determined; –, feature not present

*RoxA_35Y_ signal peptide deduced from crystal structure 4B2N and premature protein sequence. Other RoxA and RoxB signal peptides were deduced from SignalP4.1 server. Signal peptides from Lcps were deduced from TatP 1.0 server

**P_i_, isoelectric point, estimated via ExPASy compute pI/Mw tool

### Distribution of RoxA homologues

**Fig. 1:**
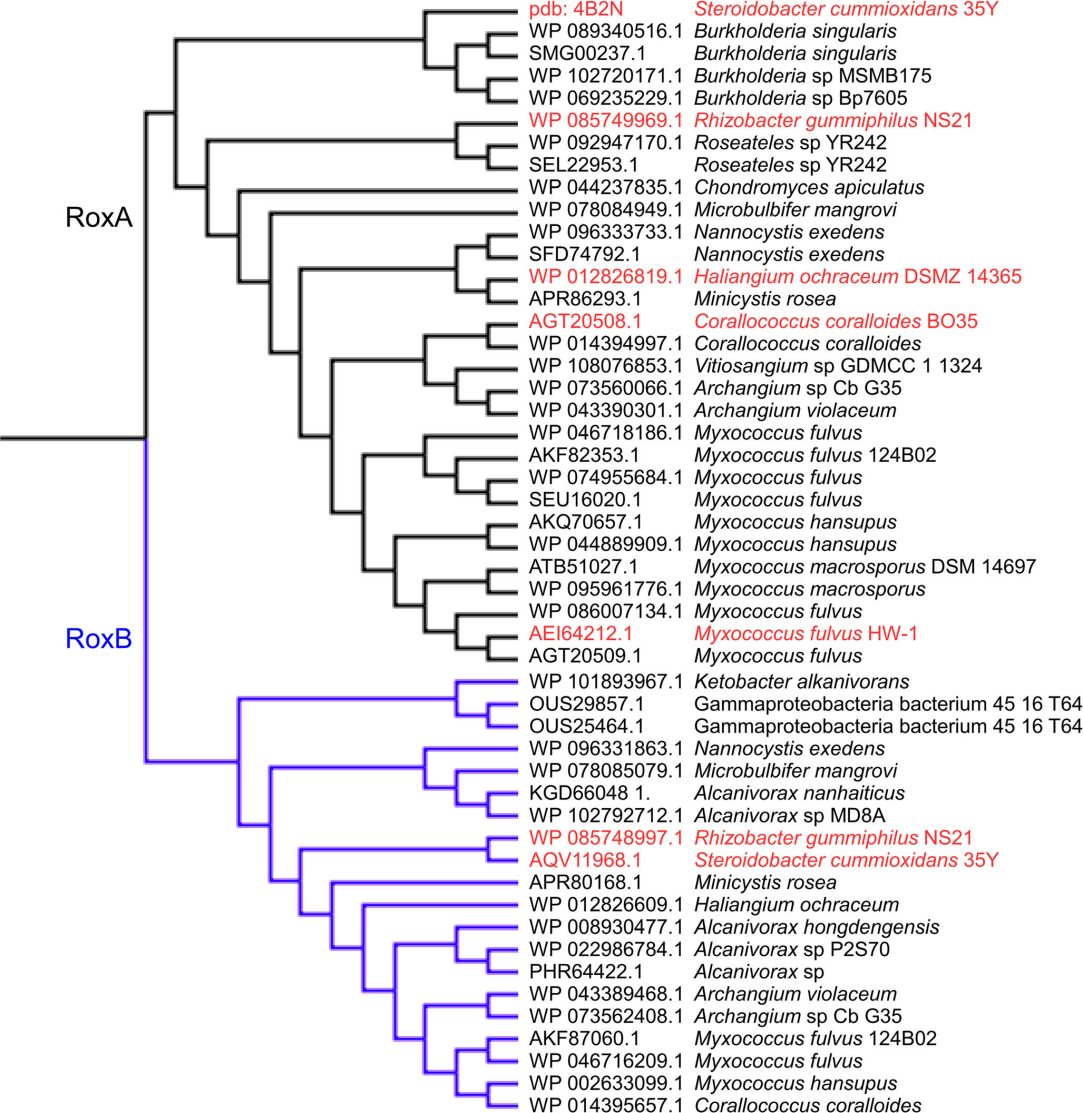
Phylogenetic relationship of rubber oxygenases of Gram-negative rubber-degrading species. A multiple sequence alignment of biochemically characterised (red letters) and postulated (black letters) rubber oxygenases was performed using the alignment software MAFFT version 7 and visualised with Archaeopteryx.js. The black branch refers to RoxAs and the blue branch indicates RoxBs. The sequences were identified using a BLASTP search with the RoxA or RoxB sequences of *S. cummioxidans* 35Y as queries. All shown RoxA and RoxB orthologue sequences have a coverage of ≥ 90% and an identity of > 60% to the query sequence, respectively

**Fig. 2:**
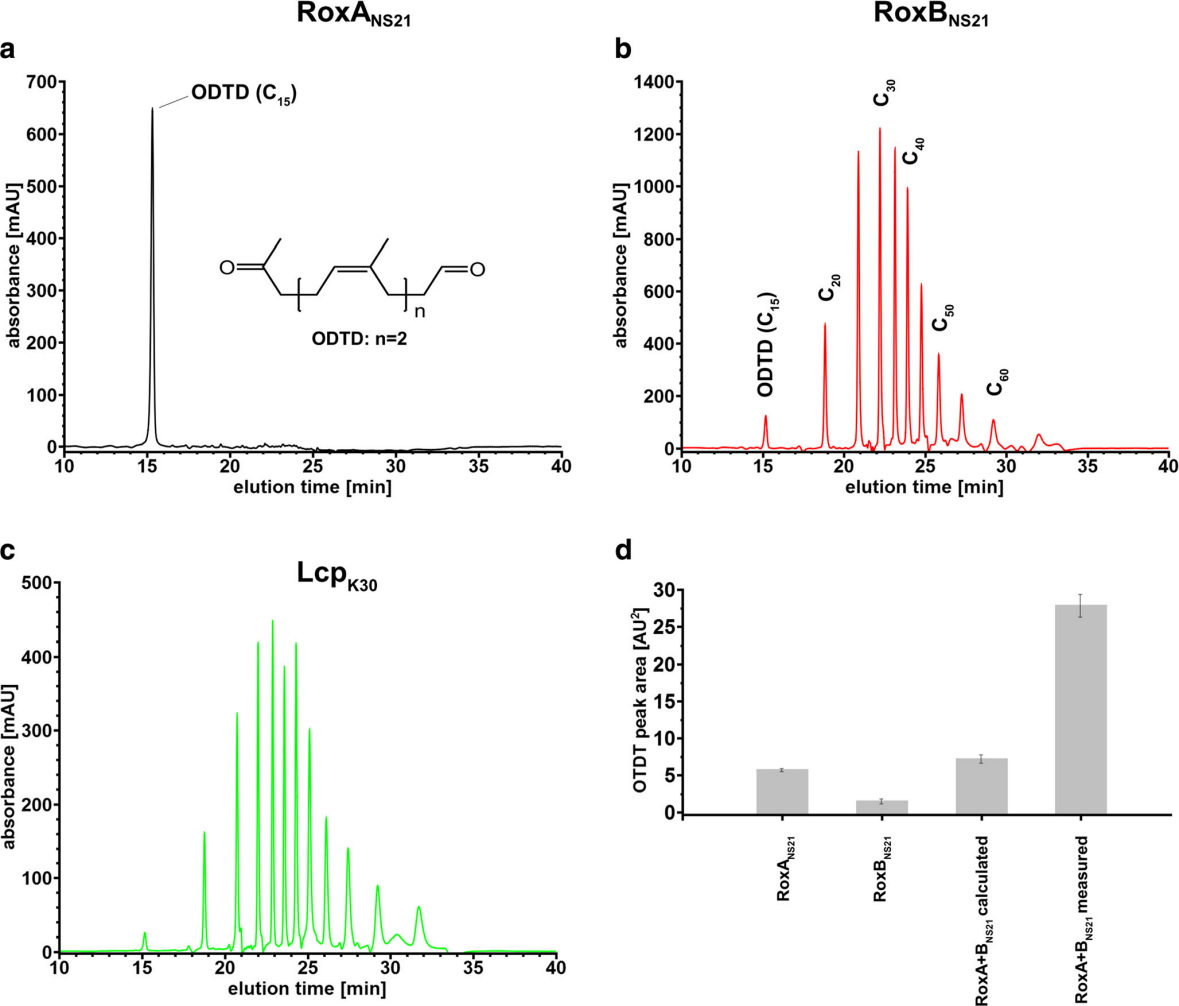
Products of polyisoprene cleavage by rubber oxygenases. Cleavage products of polyisoprene latex obtained by incubation with rubber oxygenase as indicated were analysed by HPLC as described previously (Röther et al. 2017b). RoxA_NS21_ (**a**). RoxB_NS21_ (**b**). Lcp_K30_ (**c**). Synergistic effect on ODTD formation by the simultaneous presence of RoxA_NS21_ and RoxB_NS21_ (**d**). Data from Birke et al. (2018) and Birke et al. (2015)

Since the identification of the first *roxA* gene in *S. cummioxidans* 35Y, several RoxA homologues have been identified in the translated genomes of genome-sequenced bacteria. Interestingly, RoxAs were found mainly in gamma-proteobacteria such as *Myxococcus fulvus*, *Haliangium ochraceum*, *Corallococcus coralloides* and others but recently also in the beta-proteobacterium *Rhizobacter gummiphilus* NS21 (Imai et al. 2013) (Fig. 1, Table 2). Expression, purification and biochemical characterisation of several RoxA homologues from myxobacteria (Birke et al. 2013) and from *R. gummiphilus* (Birke et al. 2018) confirmed that all of them had rubber oxygenase activity and cleaved polyisoprene to the C_15_ oligoisoprenoid ODTD as the major product, however with a significantly lower specific activity compared to RoxA of strain 35Y (Fig. 2, Table 1). No RoxA homologues have been so far detected in Gram-positive species or in *Archaea*. Therefore, it seems as if the ability to degrade rubber via a RoxA type rubber oxygenase is restricted to Gram-negative bacteria. Currently, only species of the proteobacteria have been identified as Gram-negative rubber degraders.

**Table 2:** Important rubber-degrading strains

Species/strain	Culture collection/source	Rubber oxygenase/degradation reference	Strain reference
*Steroidobacter cummioxidans* 35Y	DSMZ 103114/LMG 30900	(Braaz et al. 2004 and 2005; Schmitt et al. 2010; Seidel et al. 2013; Birke et al. 2017)	(Tsuchi et al. 1990; Sharma et al. 2018)
*Haliangium ochraceum *SMP-2	DSMZ 14365	(Birke et al. 2013)	(Iizuka et al. 1998; Fudou et al. 2002)
*Corallococcus coralloides *B035	University Hospital Bonn, Germany	(Birke et al. 2013)	(Schiefer et al. 2012)
*Myxococcus fulvus *HW1	ATCC BAA-855	(Birke et al. 2013)	(Li et al. 2002)
*Rhizobacter gummiphilus* NS21	NBRC 109400	(Kasai et al. 2017; Birke et al. 2018)	(Imai et al. 2011 and 2013).
*Streptomyces* sp. K30	University Münster, Germany	(Rose et al. 2005; Yikmis et al. 2008; Birke et al. 2015; Röther et al. 2016; Ilcu et al. 2017)	(Rose et al. 2005)
*Streptomyces griseus* 1D	University Tübingen-#3814, Germany	(Bode et al. 2001)	(Jendrossek et al. 1997)
*Streptomyces coelicolor* 1A	University Tübingen-#3813, Germany	(Bode et al. 2000)	(Jendrossek et al. 1997)
*Rhodococcus rhodocrous* RPK1	DSMZ 103064	(Watcharakul et al. 2017)	(Watcharakul et al. 2017)
*Gordonia westfalica* Kb2	DSMZ 44215	(Berekaa et al. 2000)	(Linos et al. 2002)
*Gordonia polyisoprenivorans* VH2	DSMZ 44266	(Hiessl et al. 2014; Oetermann et al. 2018)	(Arenskötter, 2001)
*Gordonia polyisoprenivorans* Kd2	University Münster, Germany	(Berekaa et al. 2000)	(Linos et al. 1999)
*Nocardia farcinica* E3	University Münster, Germany	(Ibrahim et al. 2006)	(Ibrahim et al. 2006)
*Nocardia farcinica* NVL3	Nagaoka University, Japan	(Linh et al. 2017)	(Linh et al. 2017)

### Structure of RoxA_35Y_

**Fig. 3:**
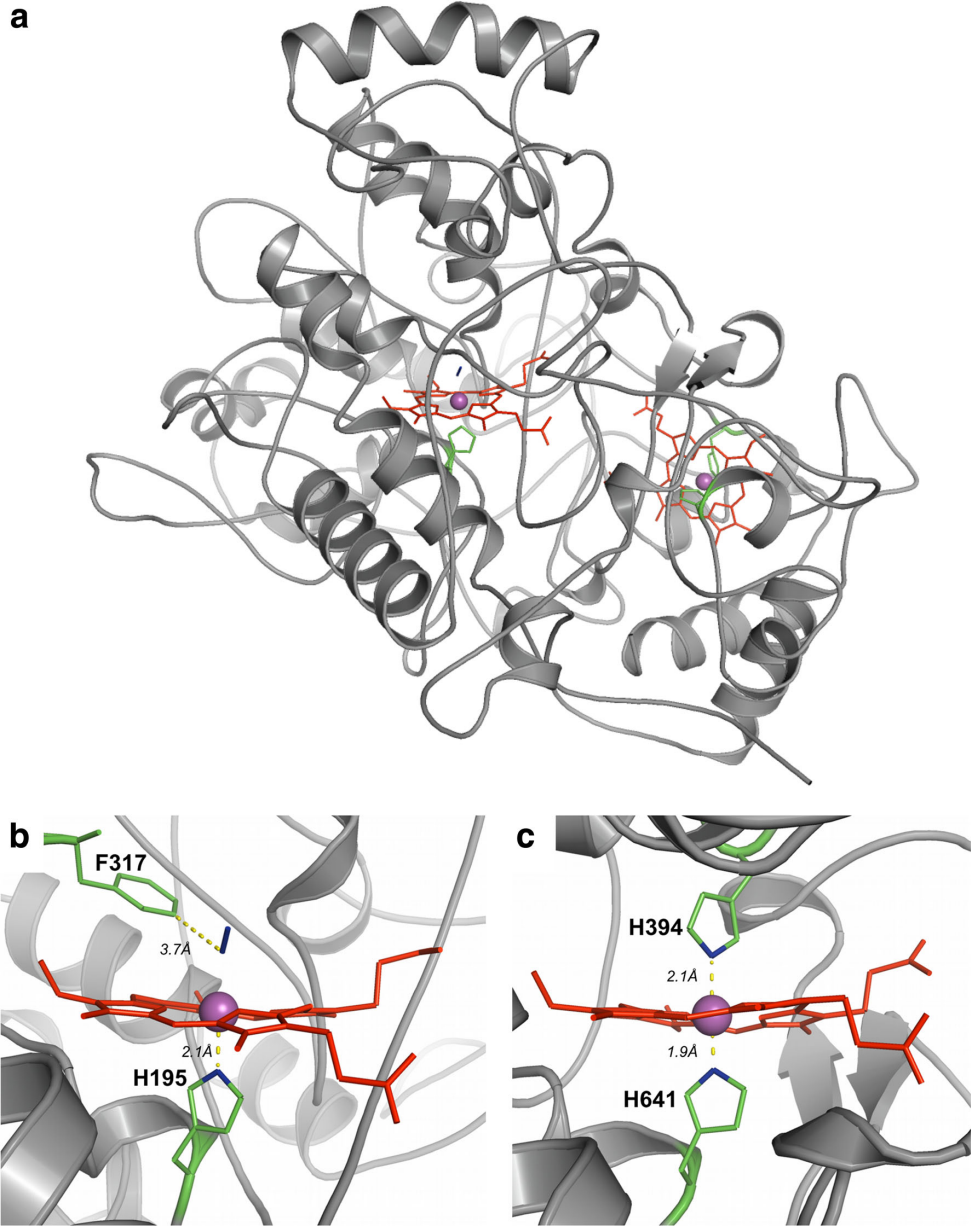
Structure of RoxA from *S. cummioxidans* 35Y. Cartoon of the structure of RoxA_35Y_ (**a**). Detailed view into the active site around the N-terminal haem (**b**). Detailed view around the C-terminal haem (**c**). Haem cofactors (red) and axial amino acid ligands (green) are shown in sticks. The central iron atom of haem is shown as a pink sphere. The dioxygen molecule bound to the haem iron is shown in blue

The structure of the 71.5 kDa dihaem rubber oxygenase RoxA of *S. cummioxidans* 35Y (RoxA_35Y_) at 1.8 Å resolution revealed a core protein comprising two *c*-type haem groups that are covalently linked to the protein via the cysteine sulphur atoms of two haem binding motifs (CSAC_194_H, CASC_393_H) (Table 1) (Seidel et al. 2013). The RoxA_35Y_ core protein has a distant structural kinship to cytochrome *c* peroxidases of the CcpA family, for example, the distances and orientations of the two haem groups are similar. However, RoxA_35Y_ is functionally different from CcpAs as it has no peroxidase activity (Schmitt et al. 2010) and is structurally different in the periphery of the molecule by the adoption of several extensions of peripheral loops resulting in a rather “large” protein (71.5 kDa) in comparison to most CcpAs (40–45 kDa). RoxA_35Y_ has an unusually low degree of secondary structures with about two thirds consisting of loops and about one third of α-helices and two short, only 3 residues comprising, β-sheets (Seidel et al. 2013) (Fig. 3a). The C-terminal haem group in RoxA_35Y_ is coordinated by two axial histidine ligands (H_394_ and H_641_) (Fig. 3c) and is less important for activity than the N-terminal haem. The latter has H_195_ as the proximal axial ligand and represents the active site of the enzyme with a dioxygen molecule stably bound as a distal axial ligand (Seidel et al. 2013) (Fig. 3b) similar to haemoglobin. A F_317_ residue in a distance of approximately 3.7 Å to the haem-bound oxygen assists in the stabilisation of the dioxygen molecule (Seidel et al. 2013; Birke et al. 2012). A substrate tunnel is not visible in the RoxA structure probably because of the hydrophobic nature of the polyisoprene substrate that would require the absence of water in a predicted substrate tunnel. It is assumed that the tunnel is formed after binding of the hydrophobic substrate molecules via flexible apolar/hydrophobic residues forming “hydrophobic brushes” (Seidel et al. 2013). Other RoxA proteins presumably are structurally very similar to RoxA_35Y_ given the high amino acid similarities of RoxAs (63 to 89% identity) and the confirmed rubber oxygenase activity of some of them (Table 1) (Birke et al. 2013) (Birke et al. 2018).

### Rubber oxygenase B

Recently, a novel type of rubber oxygenase gene with distant relationship to *roxA* genes was discovered in *S. cummioxidans* 35Y and in *Rhizobacter gummiphilus* NS21 sharing amino acid similarities of the gene products of 83% to each other but only of around 36% to currently known RoxAs. The genes were designated as *roxB*_*35Y*_ for *S. cummioxidans* 35Y (Birke et al. 2017) and *latA* in the case of *R. gummiphilus* NS21 (Kasai et al. 2017). Based on the high similarities of the properties of the purified RoxB_35Y_ and LatA protein, it became obvious that RoxB_35Y_ and LatA are homologues but substantially differ from all currently known RoxAs in some (but not all) properties. It was therefore suggested to rename LatA as RoxB_NS21_ (Birke et al. 2018). Strikingly, rubber oxygenase B (RoxB) homologues with molecular masses of around 70 kDa and with amino acid sequence identities of 60–83% to each other were present in other Gram-negative species for which a *roxA* gene was present in the genome sequence. Furthermore, the amino acid sequences of these RoxB proteins include two typical binding motifs (CxxCH) for covalent attachment of *c*-type haem groups, a conserved F_317_ homologue and a MauG motif as present in all currently known RoxAs (Table 1) (Birke et al. 2013). These findings suggest that the characterised and predicted RoxB proteins represent a separate subgroup of RoxA homologues (see also Fig. 1). Indeed, the expression of *roxB* of *S. cummioxidans* 35Y and characterisation of the recombinantly expressed and purified protein revealed that RoxB oxidatively cleaved polyisoprene at a high specific activity of ≈ 6 U/mg (Birke et al. 2017). One unit of rubber oxygenase activity corresponds to the consumption of 1 μmol dioxygen per min (for assays of rubber oxygenase activity see (Hiessl et al. 2014; Röther et al. 2017b). In contrast to RoxA_35Y_, no evidence for a bound dioxygen molecule in the *as isolated* state was found for RoxB_35Y_. The most remarkable result was, however, the finding that RoxB of *S. cummioxidans* 35Y cleaved polyisoprene randomly to a mixture of C_20_ and higher oligoisoprenoids with terminal aldehyde and keto-functional groups (Fig. 2b), a finding that was recently confirmed for RoxB of *R. gummiphilus* NS21 (Birke et al. 2018). ODTD, the C_15_ oligoisoprenoid main cleavage product of RoxA-mediated rubber degradation, was formed only in minor amounts by RoxB_35Y_ or RoxB_NS21_. These results indicated that RoxBs cleave rubber in an *endo*-type reaction. Based on the similarities and dissimilarities to RoxAs, all members of the subgroup of RoxA homologues of Gram-negative rubber-degrading species were denominated as RoxB proteins (Birke et al. 2017; Birke et al. 2018).

A remarkable consequence of the different mode of action of RoxB (*endo*-cleavage versus *exo*-cleavage of polyisoprene in RoxAs) was the identification of a synergistic effect of the simultaneous presence of both types of rubber oxygenases. The amount of the main cleavage product ODTD produced by RoxA was much higher in in vitro experiments with purified RoxA_35Y_ when RoxB_35Y_ was simultaneously present in comparison to the amount of ODTD produced by RoxA_35Y_ alone (Birke et al. 2017). This finding can be well explained by the formation of free poly/oligoisoprenoid chain ends by the action of RoxB_35Y_ that in turn increases the efficiency of the RoxA_35Y_-mediated formation of ODTD: natural rubber produced by *H. brasiliensis* has a high molecular mass of about a million. As a consequence, the number of polyisoprene molecules and polyisoprene chain ends at the surface of rubber particles is rather low in comparison to the number of molecules at the surface of low molecular compounds. Since all currently known RoxA proteins cleave polyisoprene processively from the molecules ends (see above), RoxAs must find and bind to a free polyisoprene chain end to start the cleavage reaction. Because of the low number of polyisoprene chain ends in high molecular weight rubber materials, the efficiency of rubber cleavage by RoxA alone is rather low. However, *S. cummioxidans* 35Y is one of the fastest growing strains when cultivated on rubber latex (Tsuchii and Takeda 1990) suggesting that *S. cummioxidans* 35Y has more than only one rubber oxygenase and/or the identification of RoxB_35Y_ can explain how the efficiency of RoxA_35Y_-mediated polyisoprene cleavage is improved. In the case that RoxB_35Y_ would be the only rubber-cleaving enzyme, this would require the uptake of large isoprenoid molecules with a variable number of isoprene units and this might be difficult for the cells. The synergistic effect of the simultaneous presence of RoxA_35Y_ and RoxB_35Y_, however, enables *S. cummioxidans* 35Y to convert rubber extracellularly at high efficiency to just one cleavage product of defined length (ODTD) that can be taken up by only one transport protein and can be used as a source of carbon and energy. Since RoxA_35Y_ and RoxB_35Y_ were shown to be simultaneously expressed in *S. cummioxidans* 35Y during growth on polyisoprene latex (Birke et al. 2017), the relatively fast growth of *S. cummioxidans* 35Y on polyisoprene as sole source of carbon and energy can be well explained.

The synergistic action of the two rubber oxygenases of *R. gummiphilus* NS21 (RoxA_NS21_ and RoxB_NS21_) was recently experimentally confirmed (Birke et al. 2018). This species is also able to grow on polyisoprene latex as the sole source of carbon and energy and to form clearing zones on opaque latex agar (Imai et al. 2011; Imai et al. 2013). A *latA* gene was independently identified to be involved in utilisation and cleavage of rubber, but the corresponding LatA protein was not purified and characterised (Kasai et al. 2017). Very recently, it became clear that LatA represents a RoxB homologue and that *R. gummiphilus* NS21 harboured a second rubber oxygenase, RoxA_NS21_. The RoxA_NS21_ and RoxB_NS21_ (=LatA_NS21_) proteins of *R. gummiphilus* NS21 are highly similar to RoxA_35Y_ and RoxB_35Y_ of *S. cummioxidans* 35Y (Birke et al. 2018) (see Fig. 1 for phylogenetic relationship of RoxAs and RoxBs) and also cleaved polyisoprene synergistically to ODTD as end product. Notably, when the isolated rubber cleavage products generated by RoxB_NS21_ were used as substrate for RoxA_NS21_, a subsequent HPLC analysis of the products revealed the appearance of a high ODTD peak (Birke et al. 2018). This clearly demonstrated that RoxA_NS21_ is able to use the products of RoxB_NS21_ as substrate and confirmed the synergistic effect of the two enzymes. Remarkably, almost all genome-sequenced Gram-negative bacteria that have a *roxA* gene also have a *roxB* homologue in their genome suggesting that the conjointly appearance of RoxA and RoxB homologues is a common feature of Gram-negative rubber-degrading species. This suggests that all *roxA* and *roxB* harbouring Gram-negative species will be potent rubber-degrading bacteria (clear zone formers on opaque latex agar) and will take advantage of the synergistic effect of the simultaneous expression of *roxA* and *roxB* homologues on the cleavage of polyisoprene to ODTD as a major cleavage product.

### Latex clearing protein

Despite the large number of isolated Gram-positive species with rubber-degrading capabilities (Tsuchii et al. 1985; Heisey and Papadatos 1995; Jendrossek et al. 1997; Imai et al. 2011; Yikmis and Steinbüchel 2012a; Chia et al. 2014), none of the currently genome-sequenced Gram-positive species has a *roxA* or a *roxB* gene. This indicates that Gram-positive rubber-degrading species must have a different type of rubber-cleaving enzyme. The first described gene involved in rubber degradation was identified by mutant and complementation analysis of *Streptomyces* sp. K30, a strain which produces large clearing zones during growth on opaque polyisoprene latex agar (Rose et al. 2005; Yikmis et al. 2008; Yikmis and Steinbüchel 2012b). The transfer of this gene to a mutant specifically defective in clearing zone formation during growth on rubber latex or the transfer to a non-rubber-degrading species of the genus *Streptomyces* restored or conferred the ability to form clearing zones around the arising colonies during growth in the presence of opaque polyisoprene latex. This gene was denominated as the latex clearing protein (Lcp) gene (Rose et al. 2005). The *lcp* gene of *Streptomyces* sp. K30 codes for a protein of ≈ 43 kDa (Lcp_K30_) and the amino acid sequence of Lcp_K30_ reveals no similarities to RoxAs, RoxBs or any other enzyme with known function. For the biochemical properties of Lcps and the structure of Lcp_K30_, see the chapter below.

Since the beginning of this century, the number of genome-sequenced prokaryotes has largely increased. Bioinformatic analysis of these genomes revealed an enormously high number of more than 1000 *lcp* genes among the Gram-positives suggesting that the ability to utilise polyisoprene compounds is largely distributed among the Gram-positive species. Interestingly, Lcp genes are present in species that either form (e.g. *Streptomyces* sp. K30, (Rose et al. 2005)) or do not form clear zones on latex agar (Hiessl et al. 2012; Watcharakul et al. 2016). It seems that the synthesis of an Lcp protein is an essential but not the only factor required for the formation of a clearing zone in Gram-positive rubber-degrading species. Not even one Lcp homologue is present in currently genome-sequenced Gram-negative species or in *Archaea* (August 2018). The strictly separated appearance of either RoxAs/RoxBs in Gram-negative or of Lcps in Gram-positive rubber-degrading species suggests that the ability to cleave polyisoprene evolved independently at least twice.

### Properties of Lcps

The Lcp proteins of *Streptomyces* sp. K30, *Gordonia polyisoprenivorans* VH2, *Rhodococcus rhodochrous* RPK1 and of *Nocardia farcinica* NVL3 represent the currently four Lcps that have been purified (Birke and Jendrossek 2014; Hiessl et al. 2014) (Watcharakul et al. 2016; Linh et al. 2017; Oetermann et al. 2018) (status of August 2018); the purified Lcps of *Streptomyces* sp. K30 (Lcp_K30_), *G. polyisoprenivorans* VH2 (Lcp1_VH2_) and *R. rhodochrous* RPK1 (Lcp_RPK1_) have been biochemically characterised in detail. Lcps have a molecular mass of ≈ 40 to ≈ 46 kDa and thus have roughly only half of the molecular masses of RoxAs or RoxBs (≈ 70–75 kDa). Lcps are not related in amino acid sequence to RoxAs or RoxBs. In contrast to RoxAs and RoxBs that are *c*-type dihaem proteins, Lcps harbour only one, non-covalently bound *b*-type haem cofactor as the active site (Birke et al. 2015; Watcharakul et al. 2016; Oetermann et al. 2018). Previous reports on the putative absence of a metal cofactor in Lcp_K30_ (Birke and Jendrossek 2014) or the presence of copper as a cofactor of Lcp_VH2_ (Hiessl et al. 2014) were presumably based on insufficient detection limits or artificial binding of metal ions to the protein. Lcps presumably are also involved in the utilisation of poly(*trans*-1,4-isoprene) (gutta-percha) (Luo et al. 2014).

**Fig. 4:**
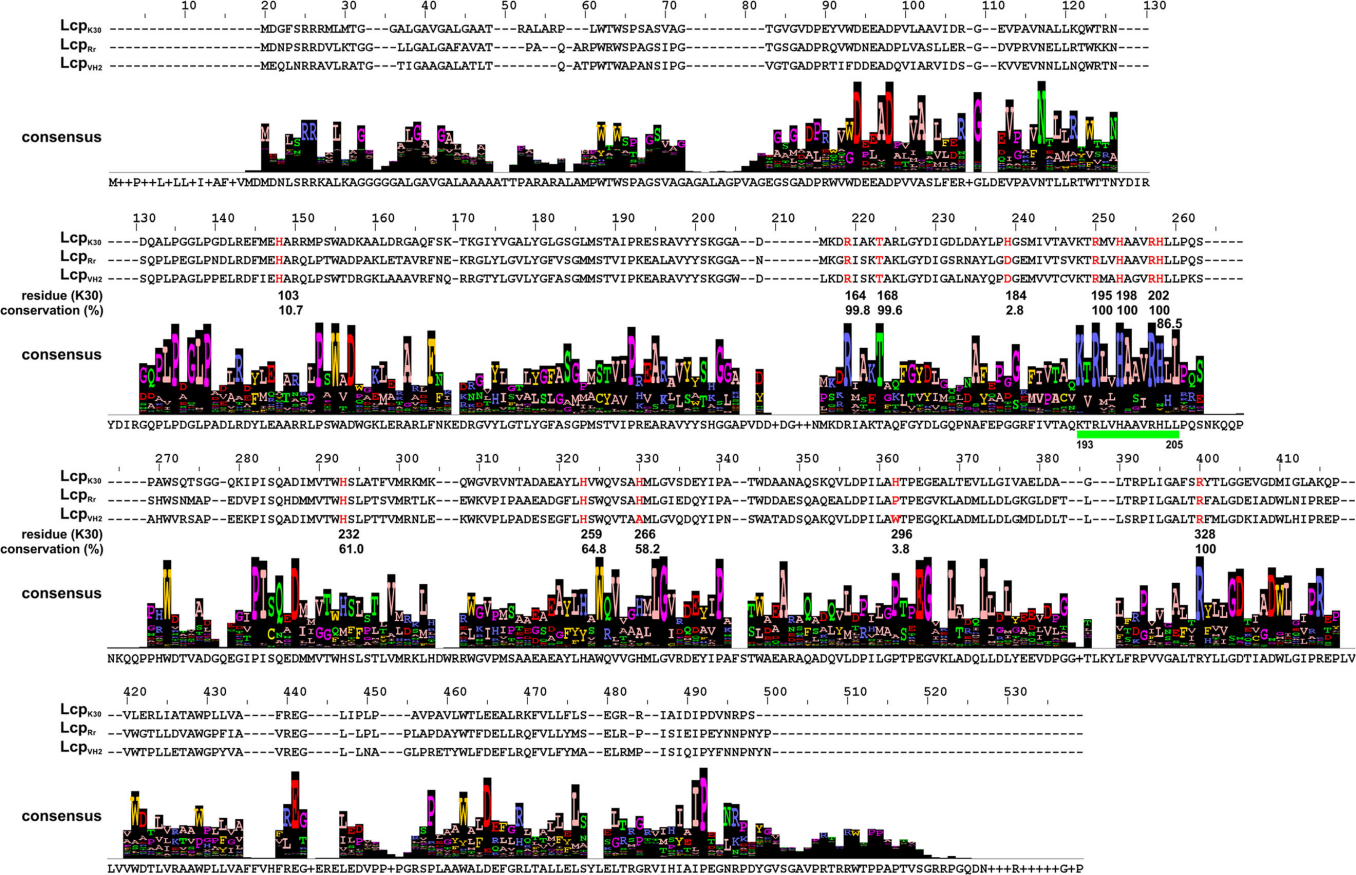
Alignment of biochemically characterised Lcps. Amino acid sequence alignment of Lcps of *Streptomyces* sp. K30, *R. rhodochrous* RPK1 and Lcp1 of *G. polyisoprenivorans* VH2. Below the alignment, a consensus sequence of 495 Lcp sequences taken from the database is shown. The height of the columns indicates the degree of conservation. The values of conservation (as percentages) for selected strongly conserved residues (highlighted in red) are given below. A 13-residue-long conserved region is indicated by a green bar. Taken from Röther et al. (2016)

The three biochemically well-characterised Lcps and a selection of 492 putative Lcp homologues from the database shared the presence of a conserved domain of unknown function (DUF2236) (Hiessl et al. 2014; Röther et al. 2016). Three amino acid residues of the DUF2236 sequence were almost invariant. These were R_164_ (conserved in 98.8% of 495 Lcp homologues, numbering according to Lcp_K30_), T_168_ (99.6%) and H_198_ (100%) (Röther et al. 2016) (Fig. 4). H_198_ of Lcp_K30_ (and H_195_ of Lcp1_VH2_ (Oetermann et al. 2018)) were identified as the proximal axial haem ligand and R_164_ and T_168_ were located close to the distal axial haem ligand K_167_ ((Ilcu et al. 2017), see below). Residues R_164_ and H_198_ (H_195_ of Lcp1_VH2_) were essential for activity and T_168_ was almost essential (2% residual activity of a T_168_A variant) for activity. Since all three residues participate in the ligation or positioning of the haem in Lcp_K30_, all other proteins with a DUF2236 domain presumably also harbour haem as a cofactor and many of the DUF2236 domain-containing proteins probably might have rubber oxygenase activity.

### Cleavage of poly(*cis*-1,4-isoprene) by Lcps

All three biochemically characterised Lcps oxidatively cleave polyisoprene randomly to a mixture of oligoisoprenoids with terminal keto and aldehyde groups. Solvent extraction of the polyisoprene latex cleavage products with ethylacetate or methanol gives C_20_ to ≈ C_65_ oligoisoprenoids that can be well separated qualitatively by HPLC (Birke et al. 2015; Röther et al. 2016; Röther et al. 2017b) or quantitatively by FPLC (Röther et al. 2017a) (Fig. 2c). The oligoisoprenoids produced from polyisoprene by Lcps are structurally identical to those produced by RoxBs (see above, Fig. 2). However, Lcps and RoxBs are not related proteins. They largely differ from each other in molecular masses, amino acid sequence and cofactor type/content (see above). The C_15_ oligoisoprenoid ODTD, the main product of RoxA-catalysed polyisoprene cleavage, was identified only in trace amounts in the Lcp_K30_ catalysed reaction. Remarkably, extraction of the Lcp1_VH2_ derived degradation products with pentane/trichloromethane or other solvents and analysis via GPC and subsequent MALDI-ToF or ESI-MS after derivatisation with Girard-T reagent (Ibrahim et al. 2006; Andler et al. 2018b; Andler et al. 2018a) showed that also very large oligoisoprenoids with > 100 carbon atoms were produced by Lcp1_VH2_. Together, these data show that Lcps cleave polyisoprene randomly in an *endo*-type fashion into products of variable length, similar to RoxBs.

### Structure of Lcp_K30_

**Fig. 5:**
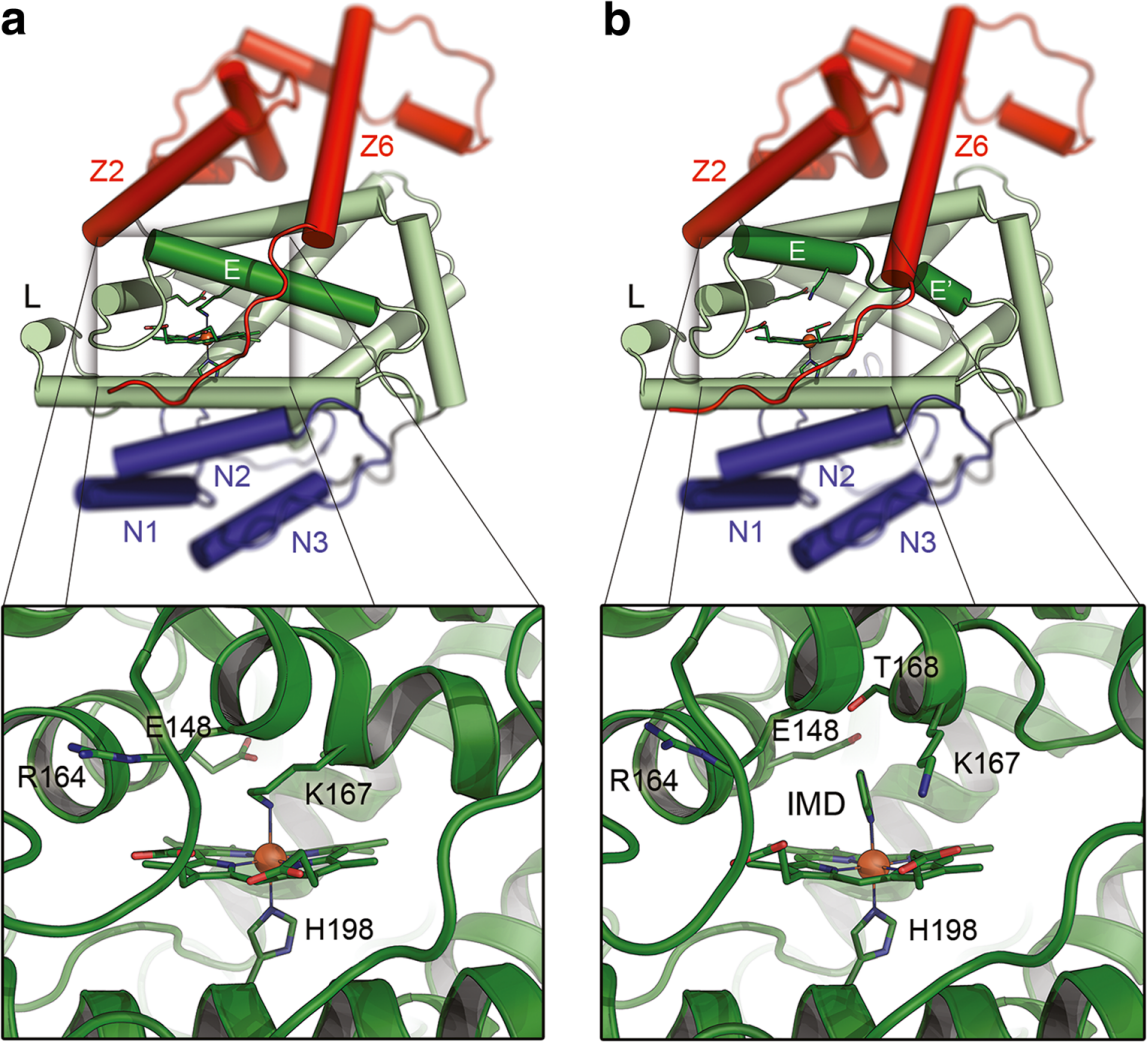
Structure of Lcp_K30_. Cartoons of the Lcp_K30_ structure in closed (**a**) and open (**b**) form are shown. The porphyrin ring and axial ligands of the central iron are shown as sticks. The Lcp_K30_ core (in green) exhibits a globin fold (helices A-H, and Lcp-specific L-helix, helix D is absent). Lys_167_ serves as a distal axial ligand to the haem group, effectively preventing access for the substrate O_2_ in the closed structure (**a**). The N-terminal extension with helices N1-N3 is shown in blue, the C-terminal extension with helices Z1-Z6 in red. In the presence of imidazole, an open form of Lcp_K30_ was obtained (**b**) in which Lys_167_ is replaced by imidazole as a distal axial haem ligand and helix E is split into two fragments. Taken from Ilcu et al. (2017)

The Lcp protein of *Streptomyces* sp. K30 (Lcp_K30_) is currently the only rubber oxygenase of a Gram-positive species with a known 3D structure (Ilcu et al. 2017). Because of the amino acid sequence similarities of Lcp_K30_ to the Lcps of *G. polyisoprenivorans* VH2 (50%) and *R. rhodochrous* RPK1 (57%), we assume that the two other biochemically characterised Lcps and many other, yet uncharacterised Lcps, have similar structures as Lcp_K30_. The majority of the Lcp_K30_ protein (63%) has an α-helical structure while the other 37% consist of connecting loops (Fig. 5). β-strands or disulphide bridges are absent (no cysteine present in Lcp_K30_). The core of Lcp_K30_ has a classical globin fold consisting of eight helices named A-H as in haemoglobin. Helix D is missing but an additional short helix (L-helix, for Lcp-specific helix) is present between helix E and F. The globin core of Lcp_K30_ embeds the *b*-haem moiety and reveals high similarities to myoglobin and to the globin-coupled sensor protein of *Geobacter sulfurreducens* (Pesce et al. 2009). Two additional domains consisting of three (N1-N3) or six (Z1-Z6) α-helices are present in Lcp_K30_ at the N-terminus and the C-terminus, respectively, and form cap-like structures at opposite sites of the globin core (Fig. 5). The specific functions of these additional domains in polyisoprene cleavage are unknown.

Two structures of Lcp_K30_ were determined, one corresponded to the closed state and one represented an open state of the enzyme (Ilcu et al. 2017). The haem group of Lcp_K30_ in the closed structure is ligated by H_198_ and by K_167_ as axial ligands. The presence of a lysine as an axial haem ligand is rare in *b*-type cytochromes. Interestingly, a slightly different structure of Lcp_K30_ was determined when imidazole was present in the crystallisation buffer and pointed to a structural flexibility of Lcp_K30_. In this case, an imidazole molecule of the solvent adopted the place of K_167_ while the spatial orientations of K_167_ and T_168_ were changed (Fig. 5b). The imidazole-bound structure showed an increased accessibility of the haem group, opening a direct access pathway to a cavity at the distal side of the haem group and despite the presence of imidazole this structure is considered to represent an open form of Lcp_K30_.

**Fig. 6:**
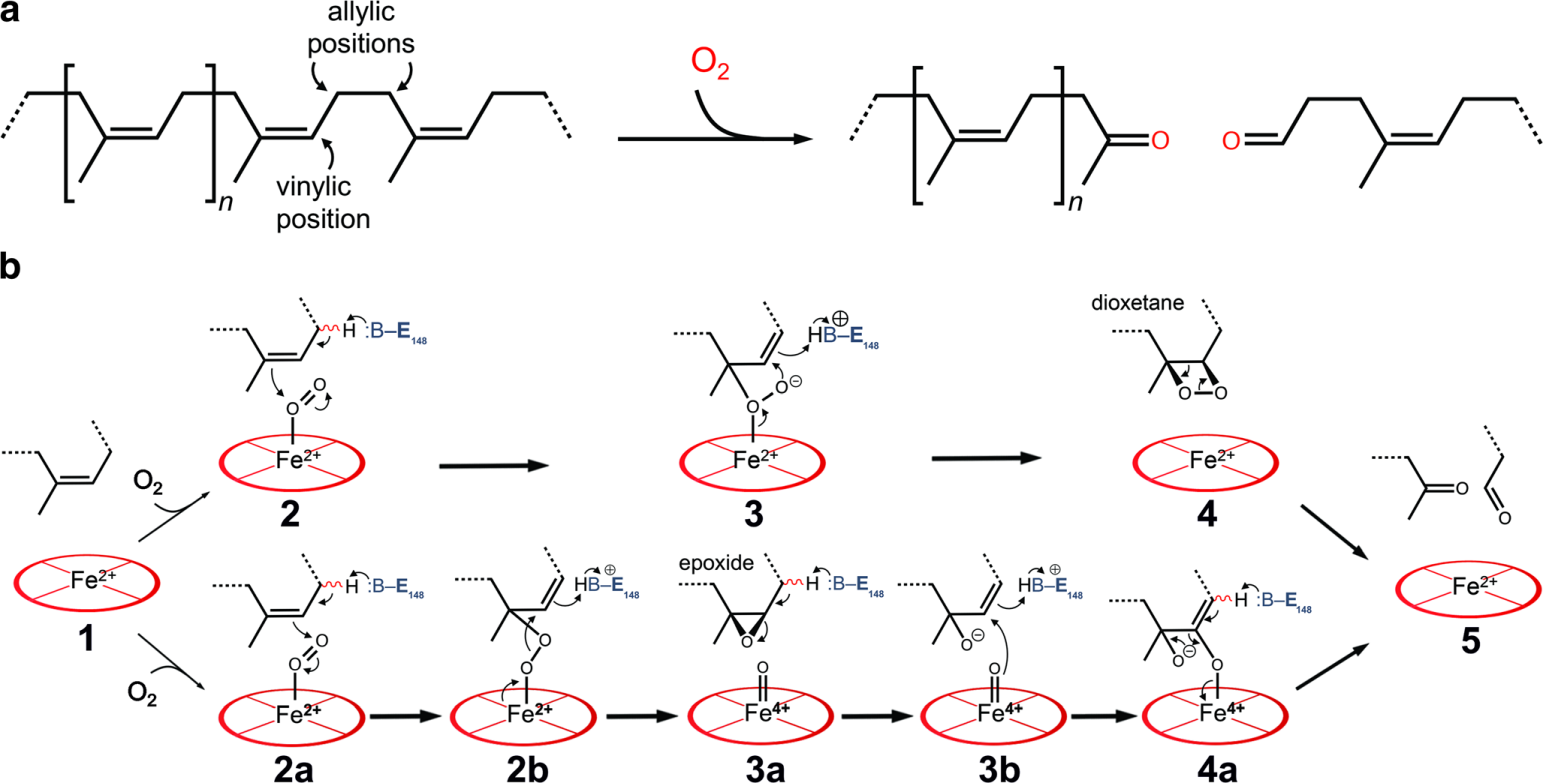
Mechanistic models of oxidative polyisoprene cleavage by Lcp_K30_. Protons in allylic positions of poly(*cis*-1,4-isoprene) will be more acidic than those in vinylic positions (**a**). LcpA_K30_ catalyses the cleavage of the isoprenoid by inserting both oxygen atoms of an O_2_ molecule. Possible reaction mechanisms (**b**). Top: the substrate polymer is threaded into the channel of Lcp_K30_ in the open state (*1*). O_2_ binds to the distal axial position of haem iron and a base in the channel, likely Glu148, abstracts a proton from an allylic position, leading to bond formation to an oxygen (*2*). The second oxygen atom, with increased nucleophilic character, attacks the adjacent carbon (*3*), leading to the formation of an instable, cyclic dioxetane intermediate (*4*) that spontaneously rearranges to the cleaved product (*5*). Bottom: alternatively, the haem-bound dioxygen can be cleaved (*2b*) to give a substrate epoxide and an oxy-ferryl intermediate (*3a*). The epoxide bond is cleaved by a nucleophilic attack of the oxy-ferryl-oxygen to the epoxide carbon atom (*3b*). Cleavage of the iron-oxygen bond (*4a*) leads to a release of the haem group and of the observed cleavage products (*5*). Taken from Ilcu et al. (2017)

Two potential reaction mechanisms are currently discussed for the enzymatic cleavage of polyisoprene (see (Ilcu et al. 2017) for details, Fig. 6). However, the postulated intermediates still await for experimental verification.

### EPR analysis of rubber oxygenases

The oxidation state of the central iron atom of the haem cofactors in rubber oxygenases and their coordination states with (axial) ligands can be determined by electron paramagnetic resonance (EPR) spectroscopy (Walker 2004). Ferrous (Fe^2+^) iron is diamagnetic and therefore does not produce any EPR signals. In contrast, ferric (Fe^3+^) iron is paramagnetic and dependent on the type of ligands in the neighbourhood of haem, the EPR signals appear at characteristic magnetic fields. So far, only RoxA of *S. cummioxidans* 35Y (RoxA_35Y_) has been analysed by EPR (Schmitt et al. 2010; Seidel et al. 2013; Ilcu et al. 2017). One of the two *c*-type haem groups in RoxA_35Y_ is present in the oxidised (ferric) form and has two axial histidine ligands (H_394_ and H_641_). The signals corresponding to this C-terminal haem centre did not change regardless whether substrates (polyisoprene latex, dioxygen) were present or absent (for details see (Schmitt et al. 2010; Seidel et al. 2013)). Therefore, the C-terminal haem centre of RoxA_35Y_ seems to be unimportant for the polyisoprene cleavage reaction. In contrast, the EPR signals of the N-terminal haem centre varied in preparation-dependent forms and in their intensities suggesting that the N-terminal haem centre is the active site and can undergo ferric-ferrous transitions. Only one of the two axial haem positions of the N-terminal haem centre is occupied by an amino acid residue (H_195_). The other axial haem position is occupied by a stably bound dioxygen molecule (Seidel et al. 2013). Lcp of *Streptomyces* sp. K30 (Lcp_K30_) is the only Lcp for which a detailed EPR analysis has been performed (Ilcu et al. 2017). In contrast to the active site of RoxA_35Y_, the (single) *b*-type haem group of purified Lcp_K30_ in the *as isolated* state is free of bound dioxygen and is present in the ferric form. However, EPR measurements revealed the presence of two distinguishable haem species: one set of EPR signals corresponded with typical signals of a *low spin* ferric iron species while the other EPR signals corresponded to *high spin* ferric signals. Remarkably, the *high spin* signal disappeared upon the addition of a typical haem ligand such as imidazole; accordingly, in the presence of imidazole, only *low spin* ferric EPR signals were recorded. This indicated that Lcp_K30_ in solution is present in two conformations: one conformation corresponds with the closed state of Lcp_K30_ in which the haem cofactor has two axial ligands (H_198_ and K_197_, see above) and produces typical *low spin* signals; the other conformation corresponds to the open state, in which the K_167_ ligation of the haem has been liberated; this haem iron with a free axial position is the source of the *high spin* signal. The fact that the *high spin* signal completely disappeared upon the addition of imidazole confirms the accessibility of the haem group by external ligands and identifies this conformation as the open state of Lcp_K30_.

### UVvis spectral properties of rubber oxygenases

**Fig. 7:**
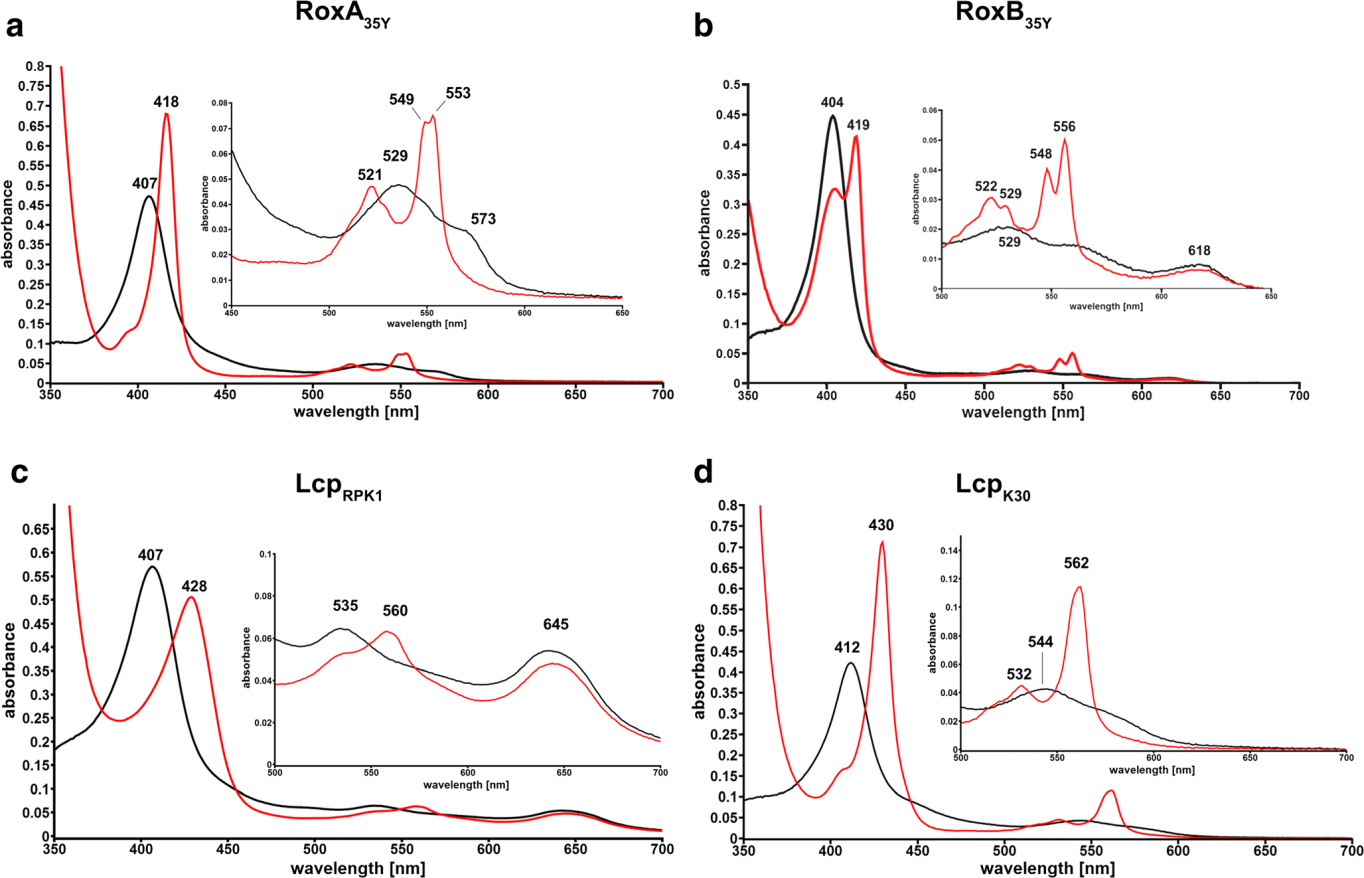
UVvis spectra of **a** RoxA_35Y_, **b** RoxB_35Y_, **c** Lcp_RPK1_ and **d** Lcp_K30_. UVvis-absorption spectra of purified rubber oxygenases as indicated were recorded for the *as isolated* (black lines) and for the chemically reduced (dithionite) proteins (red lines). In the inlay, enlarged spectra in the region of the Q-bands are shown. The numbers indicate the wavelength of the absorption maxima

Although the EPR technique is a well-suited tool to study the oxidation state and the chemical environment of haem groups, it requires the availability of expensive equipment that is not present or available in all biochemical laboratories. A more simple technique to study haem-containing proteins is to determine the absorption of these proteins in the range of ≈ 350 to 700 nm (UVvis spectroscopy). Haem-containing proteins produce characteristic spectral absorption patterns in this range (Gouterman 1961) (Fig. 7). Notably, specific absorption characteristics—dependent on which subgroup the investigated rubber oxygenase belongs to—can be recorded by UVvis spectroscopy: RoxAs *as isolated* generally produce a strong absorption at 407–409 nm (Soret band) and have minor absorptions at 529 nm (β-band) and 573 nm (α-band) in the Q-band region (Fig. 7a). A chemical reduction of RoxAs with dithionite leads to a red shift of the Soret band of about 10 nm to 417–418 nm and the appearance of split α-bands (N-terminal haem at ≈ 549–551 nm/C-terminal haem at ≈ 551–553 nm) and one β-band around 522 nm. In the presence of N-heterocyclic ligands such as imidazole or pyridine, the UVvis spectra of RoxAs resemble that of reduced RoxAs, however, with the acceptation that only the N-terminal α-band emerges (Schmitt et al. 2010). A substantial shift in the absorption of the Soret bands appears by the addition of carbon monoxide (CO) and indicates that CO can be stably bound to RoxAs *as isolated*. These features—as well as the 3D Structure of RoxA_35Y_ and EPR studies (Seidel et al. 2013)—indicate that the N-terminal haem group of RoxAs rests in a ferrous (Fe^2+^) state and firmly binds a dioxygen molecule (Fe^2+^–O_2_----Fe^3+^–O_2_^−^) that can be replaced by CO similar as in haemoglobin.

The Soret bands of the two so far purified and biochemically characterised RoxBs have a 3–5 nm blue-shifted absorption maximum at ≈ 404 nm in the *as isolated* state compared to their RoxA counterparts (Schmitt et al. 2010; Birke et al. 2013; Birke et al. 2017; Birke et al. 2018) (Fig. 7b). Additionally, weak absorptions at 529 (β-band), 562 (α-band) and 618 nm are detected, the last presumably arises due to a charge transfer (Pond et al. 1999) from a so far unknown residue. Dithionite-reduced RoxBs show only a partial Soret band shift to 418–419 nm and the appearance of a split-α (548, 556 nm) as well as a split β-band (522, 529 nm). Presumably, each signal represents one of the haem groups. The addition of imidazole to the reduced enzymes results in a complete shift of the Soret band to the reduced form; furthermore, the signal at 618 nm diminishes and the α-band at 548 nm shows increased absorbance. After addition of imidazole or CO-buffer to RoxBs *as isolated*, no significant changes in the UVvis spectrum occurred. The results identify RoxBs as completely oxidised (ferric) enzymes with a 5-fold coordinated N-terminal haem centre in the *as isolated* states and thus are clearly distinguished from RoxAs.

Lcp_K30_ and Lcp_VH2_ *as isolated* are characterised by red-shifted Soret bands at ≈ 412 nm compared to RoxAs and RoxBs, β-bands at 532–533 nm and α-bands around 570 nm (Fig. 7d). After reduction by dithionite, the bands shift to 430 nm (Soret band), 532–533 (β-band) and 562–564 nm (α-band). The spectra of Lcp_K30_ and Lcp_VH2_ do not change largely in the presence of imidazole neither in the *as isolated* nor in the reduced state; only a slight increase in absorbance was observed for the Q-band region for reduced Lcp_VH2_ (Oetermann et al. 2018). The addition of CO to Lcp_K30_ *as isolated* also has no effect on the UVvis spectrum, in contrast to the dithionite-reduced enzyme. This implies a ferric haem group in Lcps; furthermore, the majority of Lcp_K30_ molecules in solution seems to rest in a bi-axial ligated (closed) *as isolated* state.

Spectral analysis of the Lcp protein of *Rhodococcus rhodochrous* RPK1 (Lcp_RPK1_) reveals some distinct properties when compared to Lcp_K30_ and Lcp_VH2_ (Fig. 7c). The Soret, β- and α-band are at 407 nm, 535 nm and (weak) around 570 nm (Watcharakul et al. 2016); an additional feature is present around 645 nm. The addition of CO leads to detectable changes of the UVvis spectrum only after reduction (as in other Lcps). After dithionite reduction, the bands shift to 428 nm (Soret), ≈ 532 (β) and 560–562 nm (α). Notably, the Q-bands of Lcp_RPK1_ are not as defined as seen for other Lcps and the presence of imidazole significantly affects the spectrum of reduced Lcp_RPK1_. These characteristics suggest that Lcp_RPK1_ has a ferric haem group with only one axial amino acid ligand and rests in an open, for ligands accessible state. This interpretation is supported by the detection of prominent changes of the EPR spectrum of Lcp_RPK1_ upon the addition of latex (unpublished data) that was never found for Lcp_K30_.

### Secretion of rubber oxygenases and incorporation of haem cofactor

The polyisoprene molecules in NR are much too large to be taken up across the cell membrane and therefore must be cleaved extracellularly into smaller molecules. The polypeptides of the rubber oxygenases of Gram-negative bacteria (RoxAs and RoxBs) have typical N-terminal signal peptides to allow a passage across the cell membrane via the *sec* system although the involvement of the *sec* system has not been demonstrated experimentally. The incorporation and covalent attachment of the two *c*-type haem cofactors of RoxAs and RoxBs happens after the transport of the polypeptide across the cell membrane in the periplasmic space. Remarkably, expression of RoxAs (and presumably also of RoxBs) in active form is not possible in recombinant *E. coli* strains. As soon as the expression of a plasmid-encoded *roxA* gene is induced (by the addition of an appropriate inducer compound), growth of *E. coli* (or of recombinant *Pseudomonas putida*) immediately slows down and stops and no or only trace amounts of RoxA protein can be detected (Hambsch et al. 2010; Birke et al. 2012). Even the additional presence of a plasmid that harbours the complete operon for cytochrome *c* maturation and that should provide the necessary proteins for posttranslational incorporation and covalent attachment of the *c*-type haem cofactor could not increase the expression of *roxA*. The function of the cytochrome *c* maturation genes was confirmed by successful expression of another *c*-type cytochrome, the dihaem *c*-type cytochrome Dhc2 from *Geobacter sulfurreducens* (Hambsch et al. 2010). A similar phenomenon was previously described for the (unsuccessful) expression of MauG in *E. coli* (Wang et al. 2003; Wilmot and Davidson 2009). RoxAs and RoxBs both have MauG motifs in their amino acid sequences. We assume that MauG (a cytrochrome *c* peroxidase-related protein with two *c*-type haems as cofactor; MauG is required for the biosynthesis of the tryptophan tryptophylquinone cofactor of methylamine dehydrogenase) and related *c*-type cytochromes with MauG motifs employ a different cytochrome *c* incorporation/maturation system that is absent or non-functional in *E. coli*. For these reasons, rubber oxygenases of Gram-negative bacteria (RoxAs and/or RoxBs) were generally expressed in *S. cummioxidans* 35Y. The chromosomal *roxA* and/or *roxB* genes were deleted and replaced by the rubber oxygenase gene of interest under an inducible promoter. This procedure allowed a reproducible expression of large amounts of active RoxAs or RoxBs (Hambsch et al. 2010; Birke et al. 2012; Birke et al. 2017; Birke et al. 2018). The *∆roxA*_*35Y*_*-roxB*_*35Y*_ double deletion mutant of *S. cummioxidans* 35Y can be obtained from the corresponding author upon request.

Lcps incorporate the *b*-type haem group intracellularly and the correctly folded, active holoenzyme is secreted via the TAT-secretion system. This has been shown experimentally for the Lcp protein of *Streptomyces s*p. K30 (Yikmis et al. 2008) and presumably accounts for many if not all other Lcps given the widespread presence of the twin-arginine motif in the signal peptides of Lcps. The overexpression of full length *lcp* from *Streptomyces* sp. K30 in *E. coli* in active form was also not successful (Yikmis et al. 2008; Yikmis and Steinbüchel 2012b) but was fairly possible in *S. cummioxidans* 35Y (Birke and Jendrossek 2014). In contrast, intracellular expression of *lcps* in recombinant *E. coli* (by removing the signal peptide) without substantial growth inhibition by the induction of *lcp* expression resulted in good yields of active protein (Hiessl et al. 2014; Birke et al. 2015; Röther et al. 2016; Andler and Steinbüchel 2017; Oetermann et al. 2018).

### Biotechnological application of rubber-degrading microorganisms and their rubber oxygenases

Despite the ubiquitous presence of rubber-degrading microorganisms in most ecosystems on earth, the biodegradation process of products with rubber as a main constituent is very slow. Even under optimal laboratory conditions, it takes weeks or even months until a substantial weight loss of solid rubber is obtained. Moreover, most rubber products contain additives such as antioxidants and other stabilisers; furthermore, the polyisoprene molecule chains of most rubber items are cross-linked by sulphur bridges (vulcanised rubber). These modifications inhibit microorganisms and/or their rubber oxygenases (Berekaa et al. 2000). Furthermore, the biodegradation process is limited to the surface of the rubber materials because neither microorganisms nor rubber oxygenases can penetrate the water-insoluble material. These drawbacks potentially can be overcome by appropriate pre-treatments of the rubber items, such as grinding to increase the available surface, solvent extraction to remove antioxidants/stabilisers, and chemical or biological treatments to break the sulphur bridges. These necessities surely will largely increase the costs of the biotechnological process. For these reasons, the simple combustion of rubber items is at present more promising compared to the sophisticated biodegradation of rubber because a costly pre-treatment is not necessary and the released heat-energy can be at least partially used for production of electricity.

On the other hand, rubber oxygenases, in particular RoxAs, are rather stable enzymes and can be used for the biotechnological production of oligoisoprenoids as fine chemicals of defined length (C_15_ to ≈ C_65_) from rubber materials. Such oligoisoprenoids are highly active due to their keto and aldehyde functionalities and can be used as building blocks in chemical reactions for the synthesis of compounds with more complex structures. For the first examples of laboratory production of oligoisoprenoids using Lcp, see Andler and Steinbüchel (2017) and Andler et al. (2018b). Individual oligoisoprenoids of defined length (C_15_ to ≈ C_65_) can be isolated from RoxB- or Lcp-derived oligoisoprenoid mixtures by FPLC as described in Röther et al. (2017a). The C_15_-oligoisoprenoid ODTD can be more efficiently obtained by a combination of RoxAs with RoxBs or of RoxAs with Lcps instead of RoxAs alone because the increase in the number of oligoisoprene chain ends by RoxB/Lcp leads to a more efficient RoxA catalysis (see part on synergistic effect above).

Last not least, rubber oxygenases could be used to “bioprint” rubber surfaces. For example, the exposure of (selected areas of) a rubber surface to rubber oxygenases will introduce keto/aldehyde functions to the rubber surface only at these selected areas that can further react (“be developed”) with other chemicals in a second step thereby creating a (potentially visible) pattern on the surface of the rubber item. For such applications, only minor amounts of rubber oxygenases and only a very limited degree of degradation of the rubber materials would be necessary.

## Outlook

The properties of the currently biochemically characterised rubber oxygenases produced by Gram-negative species (RoxAs and RoxBs) and by Gram-positive rubber degraders (Lcps) are summarised in this overview. While RoxAs specifically cleave polyisoprene to only one major cleavage products (C_15_ isoprenoid ODTD), RoxBs and Lcps cleave rubber randomly to a mixture of oligoisoprenoids of different length. Up to now (summer 2018), no example of the presence of a *roxA* gene in a Gram-positive species was identified in the database. This is surprising at least for the clear zone-forming Gram-positive rubber-degrading species as RoxAs exert a synergistic effect on the efficiency of Lcp-catalysed polyisoprene cleavage. In the case of adhesively growing rubber-degrading species, the close association of the bacteria with the polymeric substrate might compensate this disadvantage: the uptake system for rubber cleavage products is more efficient when dilution of the product concentration by diffusion is less pronounced because of the direct contact with the substrate. This might be the explanation why Gram-negative rubber degraders and Gram-positive, adhesively on rubber growing strains, grow faster on rubber than not adhesively growing Gram-positive rubber degraders.

Due to the ubiquitous presence of compounds harbouring isoprene units, it is reasonable to predict that rubber-cleaving enzymes will be also present at least in some *Archaea* and in fungi. Reports on rubber-utilising fungi are frequent but to the best of our knowledge, publications on rubber-utilising *Archaea* are missing at present. It will be interesting to determine if rubber-degrading *Archaea*/fungi also have RoxAs/RoxBs or Lcps, or if they employ different, yet undiscovered types of rubber-cleaving enzymes for the initial cleavage of polyisoprene.
